# Data quality indicators for daily life chart methodology: prospective self-ratings of bipolar disorder and alcohol use

**DOI:** 10.1186/s13104-015-1436-x

**Published:** 2015-09-24

**Authors:** Stasja Draisma, Jan van Zaane, Johannes H. Smit

**Affiliations:** Department of Psychiatry and EMGO+ Institute, VU University Medical Centre Amsterdam, A.J. Ernststraat 1187, 1081 HL Amsterdam, The Netherlands

**Keywords:** Data quality, Bipolar disorder, Life chart methodology

## Abstract

**Background:**

Self-rating instruments which require a large number of repeated assessments over time are increasingly popular in psychiatry. They are well suited to describing variations in mental states, especially in order to investigate effects of behaviour and events on functioning and mood. For bipolar disorder, the self-rating instrument ‘NIMH daily life chart’ was developed to assess the course of the illness. This instrument has been validated in the customary ways, yet information about data quality (e.g. completeness, consistency, construct validity, reactivity) was lacking. The goal of this study was to develop several data quality indicators computed from data, in order to be able to detect respondents that provide less valid or reliable data.

**Methods:**

During approximately 1 year on average, 137 patients with DSM-IV diagnosed bipolar disorder rated their mood, functioning and number of alcohol units consumed on a daily basis. Three kinds of quality indicators were developed: (1) compliance (i.e. completeness of recording on a daily basis), (2) the association between conceptually related variables—construct validity—and (3) reactivity: any changes in alcohol-drinking behaviour due to the assessments themselves. Relations were measured with Spearman’s rho.

**Results:**

A relation was found between data quality and illness severity: respondents with lower data quality, according to our operationalisations, were more strongly affected by the illness, as expressed in the number of ill days, than respondents with higher data quality.

**Conclusion:**

The more affected patients are by the illness, the lower the data quality to be expected in life chart reports.

## Background

### Assessing the course of bipolar disorder

Bipolar disorder (BD) is a serious, recurrent mental illness, characterised by mood swings that vary in their duration and frequency between manic, depressive and euthymic states. The illness has a variety of forms, with several subtypes of mania and depression (e.g. hypomania, severe mania, major depression, and minor depression). Initial diagnosis of bipolar disorder is difficult, and often occurs 5–10 years after the onset of the first symptoms [1]. This delay in diagnosis is caused by the variability of symptoms, phases and subtypes. In addition, BD is accompanied by high comorbidity of other syndromes, such as alcohol use and personality disorders. More systematic insight in such illness variations is needed to provide a good descriptive base for clinical phenomenology. This requires flexible and comprehensive research methods. The variation in illness presentations initially hampered the development of sound research designs (clinical trials, longitudinal surveys) for studying the course of BD. To overcome this problem, several authors [1–3] have argued that to study the varieties in the course of BD in a valid and reliable manner, fine-grained longitudinal assessment methods are required. To identify and compare subtle mood changes, reports are necessary with respect to the severity of symptoms and risk factors on a prospective day-to-day basis, either through patient self-ratings or by clinician evaluations [4]. For example, to study the short-term effects of changes in alcohol use on illness severity, daily observations of alcohol consumption and illness severity are required [5]. Also, prospective daily self-ratings enable the study of within-person processes over time and suffer less from recall bias as found in retrospective ratings [6]. In this manner, better understanding and management of disease development within patients with BD can be attained [2]. The choice for daily prospective monitoring, instead of hourly or weekly, is also supported by the finding that the circadian rhythm and associated variables (e.g. hours of sleep) greatly influence cycle acceleration and mood change in bipolar disorder [7]. To enable fine-grained analysis of mood swings, the Stanley Foundation Bipolar Network group used the National Institute of Mental Health prospective Life Chart Methodology (LCM), to record behaviour and illness symptoms [1, 8]. The LCM uses day-to-day self-ratings, since this allows for the description of the severity, frequency, duration and episodic patterns of affective functioning of the patient. When daily LCM ratings are used, mood swings can be recognized at an early stage and it is possible to adapt treatment to the individual disease pattern. Within the LCM, with respect to illness symptoms, each day is characterized in terms of the *severity* of manic and depressive symptoms, on the basis of mood-related functional impairment in patients’ usual educational, social or occupational roles. Moreover, other relevant context variables, such as medication use, behaviours (including consumption behaviours), feelings and life events, are recorded daily. In this manner, daily mental processes in interaction with contextual features can be studied. The inclusion of several domains enables the study of patterns of co-variation in time between different variables. Initially, the Life Chart was developed as a clinical tool to assist patients and clinicians with disease management. Gradually, it has become an important instrument for assessing BD characteristics scientifically, as expressed in a growing number of publications that feature the instrument [3, 9–14]. Good psychometric quality of the LCM was demonstrated in several studies, including interrater reliabilities of the instrument by different clinicians and high correlations with well-known cross-sectional rating scales such as the Inventory of Depressive Symptomatology-clinician-rated (IDS-C) and the Young Manic Rating scale (YMRS) [15, 16].

### Data quality of bipolar disorder assessments

This study concerns the data quality of the LCM. Data quality can be defined as the state of completeness, validity, consistency, and accuracy that makes data appropriate for a specific use. The issue of the *data quality* of self-ratings is an important one: Kessler [17] points to the assumption that self-ratings are subject to greater error than clinician judgments in diagnostic interviews. In an interview, the interviewer can serve as a tool to help respondents overcome difficulties with respect to e.g. comprehension and interpretation of concepts. Self-rating instruments like the LCM rely on respondents’ own judgments and interpretations. In the field of survey research, there is a tradition of investigating and publishing about data quality and measurement error [18–20]. However, studies on data quality of *daily* observation instruments are scarce. Some research concerning data quality has been published with respect to comparable self-rating monitoring instruments in psychiatry, e.g. the Experience Sampling Method (ESM), which can also be used to study dynamic mood changes over time and the situational determinants thereof [21], and the Ecological Momentary Assessment (EMA) [22]. These authors address several threats to data quality. For instance, lack of compliance with the research protocol may result in hoarding and backfilling, especially when paper-and-pencil instruments are used. Also, the intrusiveness of the method can result in induced, rather than recorded, experiences. Socially desirable answering may occur, especially when task difficulty is high. Daily self-ratings provide valuable, detailed information, yet the data gathering process may be hampered by specific complexities. Several *advantages* of illness data gathered using self-monitoring instruments on a prospective daily base are also reported in the literature [2]:

1. They provide illness information that is usually unavailable in a hospital or GP’s records.
2. Daily reporting reduces retrospective recall bias, since it puts less strain on remembering processes.
3. Events from different domains and contexts can be related to each other, enhancing ecological validity [23].
4. Information provided by the patient will be more complete than information in retrospective reports.

However, some *disadvantages* have been mentioned too:

1. Daily reporting of behaviour or feelings requires commitment and may be experienced as a burdensome recurrent task resulting in less *compliance*, expressed in larger numbers of item non-response, dropout or decreasing compliance rates over time, resulting in missing data [2]. As such, a longer registration period may be harmful to data quality, since it invites respondents to sloppy registration [24].
2. The task burden may be relieved by not reporting on a daily base (as required), but on estimations after a certain number of days; this is called “backfilling” [22].
3. Reactivity may occur, concerning a change in the behaviours under study, due to the measurement process itself. The awareness of behaviours or psychological states to which patients are otherwise inattentive may alter the behaviour under study in a socially desirable way [25].
4. Self-presentation tendencies such as socially desirable reporting can occur, for example underreporting of systematic alcohol use by heavy drinkers [26].

## Aim of this study

Until now no systematic empirical evaluation of data quality regarding the LCM instrument exists. The present study evaluates and compares possible data quality indicators for the LCM life chart instrument. Quality indicators are constructed and their performance and significance are evaluated, for example by identifying their effect on generally known BD illness severity variables.

## Methods

### Sample

A total of 180 outpatients were approached at 13 Dutch mental health treatment centres and at the Dutch Association for Manic-Depressive Patients and Relatives (DAMDR). Of these, 158 patients (88 %) entered baseline assessment. Subjects were asked to fill out the prospective life charts daily for a period of at least a year, during which monthly clinician visits were also planned. During these visits, clinician and patient discussed the life chart data of the previous month, after which these life chart registrations were approved. Of the 158 patients who completed the baseline assessment, 137 subjects (87 %) participated in the study for at least 2 months. The analyses in this study are based on these 137 subjects, which enables comparison of the data quality of persons with long and short registration periods. A minimal observation period of 2 months was chosen since a variation in the length of the recording period can be expected among daily-reporting studies, as reflected in compliance. An observation period of less than 2 months was considered problematic for studying variation. Only one person provided a life chart of one month, which was moreover only partially filled out. Of the 137 patients who completed two or more life charts, 124 patients (91 %) participated for at least 6 months, and 83 patients (61 %) completed at least a whole year, i.e. 365 days or more. Reasons for non-completion beyond 6 months were: aversion to the daily registrations, developing a severe depressive or manic episode, worsening of alcohol dependence, death (n = 2) and other reasons. The sample is described more extensively elsewhere [5]. The study was approved by the Medical Ethical Review Committee of the University Medical Center Utrecht (The Netherlands). All patients gave written informed consent after the aims of the study were explained to them (mainly, obtaining insight into the relation between substance use and illness course).

### Data collection: instruments

1. At entry, the Structured Clinical Interview for DSM-IV (SCID-I) was administered by trained mental health care professionals in order to establish the diagnosis of bipolar disorder in a reliable manner.
2. The LCM used for the present study is a paper-and-pencil instrument, consisting of an A3-size page on which relevant information can be recorded during a period of 31 days. Behaviour domains are: hours of sleep; alcohol, cigarette, coffee, tea and cola use; cannabis intake, and medication use. Space is available for recording important life events. Mental states are assessed by two separate scores: (1) illness severity scores (range −10 to +10) and (2) mood scores (range 0–100). The instruction to patients regarding experienced illness severity prescribes that “severity is based on your level of functional impairment due to depressive or manic mood symptoms in your usual social, educational, and occupational roles”. Hospitalization for mania or depression is rated at the most severe level of plus or minus 10. Severity reporting is set out in Fig. 1.

**Fig. 1:**
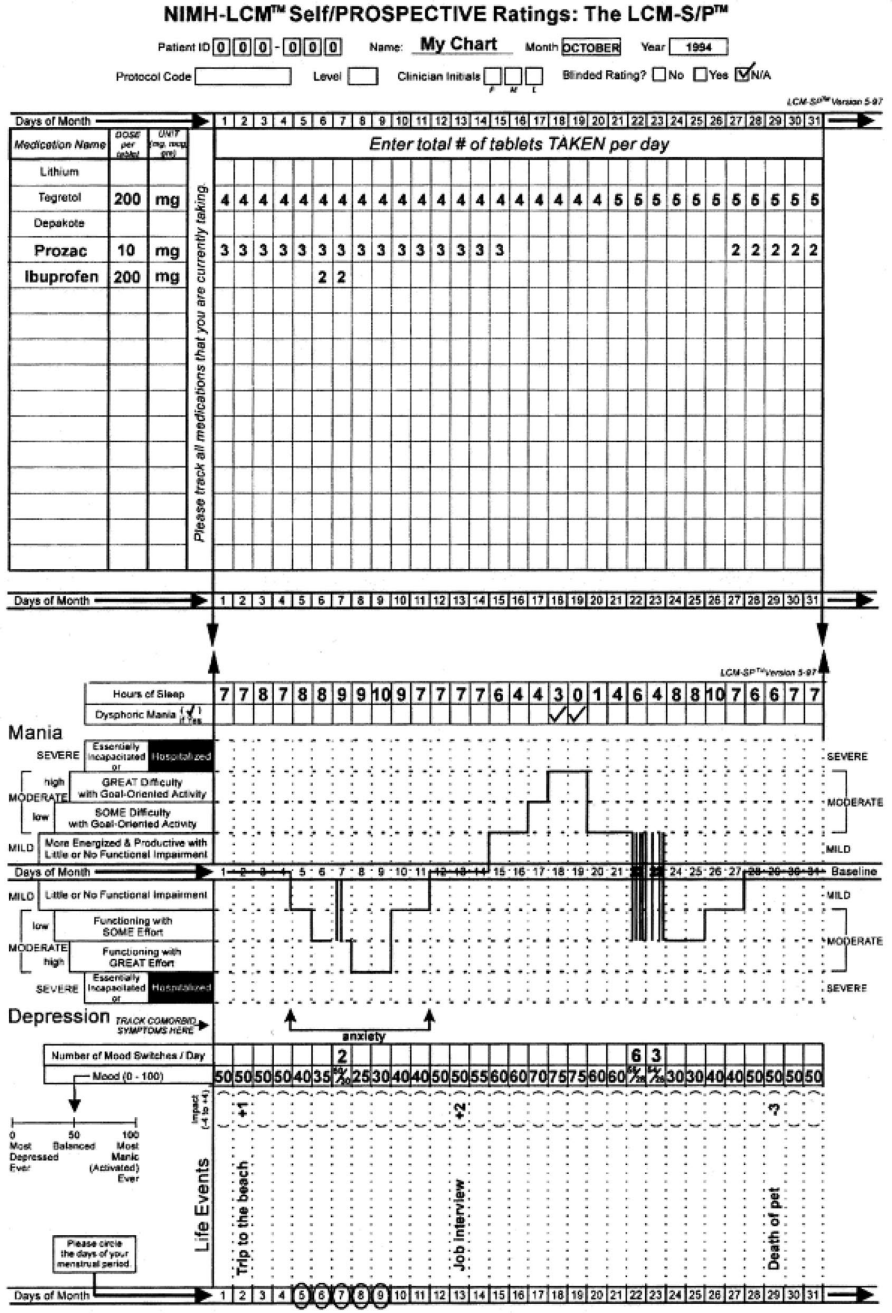
Example of aNIMH daily life chart form. Daily consumption of medicine and possibly drugs, drinks at the* top* of the chart, daily rating of functioning and mood at the *bottom*
  Episodes of depression are indicated below a baseline in the chart at four levels (mild, low moderate, high moderate or severe) and mania is indicated above these lines, using the same four levels. In the middle is the baseline, which indicates a euthymic level or balanced mood state (not depressed or manic). Daily *illness severity* scores range from 0 (euthymic or normal) through 2.5 (minimally ill), 5 (low moderate), and 7.5 (high moderate) to 10 (severely ill). Depressive symptom severity scores are indicated by negative scores, manic symptoms with positive scores. Thus, the severity variable can reach scores from minus 10 to plus 10, with intervals of 2.5. *Mood* scoring is described in the introduction as follows: “The mood scale assists you in rating your mood in fine gradations: the scale is from 0 to 100 (0 = the most depressed you could imagine being; 50 = a balanced or level mood; 100 = the most energetic/activated/manic you could ever be)”. Severity scores are supposed to indicate ‘functioning’, whereas the mood score reflects ‘feeling’. Patients were also asked to report daily on their alcohol use (number of alcohol units), for which patients received written and verbal instructions concerning the standard units of alcohol in beer, wine and spirits. Negative effects of alcohol use on BD illness severity are well documented [5]. It is therefore relevant to investigate whether a moderator effect on the relation between alcohol use and illness severity on account of data quality indicators exists. To this end, the dataset described in van Zaane et al. [5] is used to establish and investigate the effects of quality indicators.
  At baseline and at every monthly visit during follow-up, the LCM registrations were checked and approved by research assistants. Patients were requested to register their symptom severity, mood and actual alcohol use every day for a period of at least 12 months. More than half of the sample kept life charts for more than 12 months.
3. Furthermore, during monthly visits clinicians rated the functioning or illness severity of the respondents over the preceding week using the CGI-BP (Clinical Global Impression Scale-Bipolar Version [27, 28]). The CGI-BP requires the clinician to rate the severity of the disorder during the preceding week on a seven-point scale, in which 1 denotes ‘not at all ill’ and 7 denotes ‘very severely ill’. All clinicians were trained in establishing CGI scores. Mania and depression were rated separately in this CGI-BP, resulting in two separate variables. A total of 3288 CQI ratings (137 × 12 × 2) was possible. However, 14 % (N = 460) missing values were present in these ratings.

### Variables

Several types of variables were discerned:

1. *Demographic variables*, such as age at enrolment, gender and education.
2. *Clinical variables*, divided into *apparent*—i.e. directly observed—and *constructed* variables. Apparent variables are the daily reported number of alcohol consumptions, mood score, and illness severity score. Also, monthly CGI scores provided by the clinician were used as apparent variables: one for mania and another for depression, ranging from one to seven. Constructed clinical variables (used as outcome measures) are the ‘total numbers of ill days in a year’ and ‘number of illness episodes’ based on DSM IV criteria. An ill day was defined as a day with a manic illness severity score of at least mild mania (severity score 2.5 or higher) or a score of at least moderate depression (score −5 or lower). The total numbers of ill days was transformed into a proportion of the total observation period. A manic episode consisted of at least 7 days of moderate mania (score 5 or higher), a hypomanic episode of at least 4 days of mild mania (score 2.5 or higher), and a depressive episode of at least 14 days of moderate depression (score 5 or lower). The experienced numbers of episodes were corrected for number of observations.
3. *Quality indicators*, constructed using the abovementioned variables. Their definition and operationalisation are described in the next section.

### Quality indicators

For our purpose, a requirement for quality indicators is that their values can be established using the data at hand. Three data quality concepts are used in relation to the dataset obtained with the LCM life chart:

1. *Compliance* Compliance, i.e. recording on a daily basis, can be defined as a compound concept consisting of two constituents. The first constituent is the number of days for which reports are available (length of observation period). The second is the percentage of missing data (item non-response) within the observation period.
2. *Consistency* Biemer and Lyberg [18] mention internal consistency checks for the assessment of data quality, which may take several forms. In our study, two operationalisations of consistency were possible and were used.
  A strong association between conceptually related variables was expected. In the present study, this was translated into the expectation of a positive strong association between illness severity and mood ratings (i.e. severity scores and mood scores). These two kinds of self-reporting scores are recorded in the LCM daily (see Fig. 1).
  Also, judgment scores on the same variables obtained from the clinician or by means of self-rating instruments should correlate strongly [16, 29], indicating adequate construct validity. We expected a relation between the CGI-BP clinician scores on mania and depression for the preceding week obtained monthly and the daily reports for that same week by the patient.
3. *Reactive effects* These concern the fact that screening and assessment affect behaviour under study. A change in reporting of sensitive behaviour in the desirable direction as a result of the fact that the behaviour should be reported is often seen in research as well as in treatment. The evidence for a reactivity effect pertaining to alcohol use is precarious [25]. Sometimes only an effect of assessments on binge drinking or risky drinking behaviour is found, and not on overall drinking volume. Johnson et al. [2] assessed behaviour, environment, contexts and mood in an ambulatory monitoring set-up for patients with schizophrenia, anxiety or substance dependence and controls, and found that the majority of variables investigated did not change in frequency as a function of study duration. However, some evidence was found that socially sensitive behaviours—such as self-care behaviour—changed in a manner consistent with reactivity. However, in most studies the number of assessments is lower than ten and not fine-grained. In our study, which uses daily ratings, it may be expected that the initial registration of alcohol use will suffer from reactivity: some respondents may report less alcohol use after the first week of alcohol reporting, as a result of growing awareness of their alcohol consumption. However, this reactivity is expected to diminish after a while, due to the rather long duration of the recording period and the assumed chronic nature of patterns of alcohol consumption. Patients will probably not be able to maintain lower levels of alcohol consumption than are usual for them for such a long period.

### Procedures for data quality indicators

For each indicator, values in the continuum between zero (extremely low data quality) and one (perfect data quality) are established per case. This enables comparison between indicators. To accomplish this, the following general transformation algorithm for the raw quality indicator scores was used: transformation from range Xmin to Xmax to a range of Ymin to Ymax: Yi = [(Xi − Xmin)/(Xmax − Xmin)] × (Ymax − Ymin) + Ymin. Ymin takes on the value of 0, and Ymax the value of 1, whereas the X values are the original values of the indicators.

#### Compliance

(A) Using the transformation algorithm, the lengths of the individual observation periods were established and transformed into scores ranging from 0 (shortest observation period, 62 days) to 1 (longest observation period, 529 days). As an illustration, the algorithm used for the observation period is: index value = [(individual observation period − 62)/(529 − 62)]*[(1 − 0) + 0]. The result for an observation period of 100 days would be 0.08 and for 200 days 0.3.
(B) The ‘missing observations percentage’ could be established for the whole observation period (observed range: 0–30 %). The percentages were transformed into a scale from 0 (largest percentage of missing observations within the whole observation period) to 1 (no missing observations).

#### Consistency

(A) A correspondence between daily mood scores and daily severity ratings was expected. Individual correlations between the daily mood and severity scores were calculated and translated into a scale from 0 (lowest correlation) to 1 (perfect correlation).
(B) During monthly clinician visits, illness severity was established using the CGI. The severity of depression score—ranging from 1 (normal, not at all ill) to 7 (extremely ill)—and the severity of mania score were used. Patients’ daily ratings of mania and depression severity were averaged for the same seven weekdays as those on which the clinician score was based. Correlations were calculated between the scores of the total series of monthly visits in which CGI scores were established and the series of severity scores of the weeks preceding the visits, if available. Individual correlations were transformed to a scale from 0 (lowest correlation between clinician judgment and self-report) to 1 (perfect correlation between clinician judgment and self-report), using the algorithm mentioned above.

#### Reactivity

Behaviour change due to the measurement process itself was operationalised as follows. The average number of alcoholic drinks for week number 4 was subtracted from those for week number 1. Positive differences indicate a reactivity effect: the person has diminished the number of drinks consumed. Negative differences indicate an increase in consumed alcohol. The largest positive difference was translated into 0 (maximum reactivity), and the largest negative difference into 1 (least reactivity).

### Statistical analyses

For summary statistics, means and standard deviations were calculated for continuous variables; counts and percentages for discrete variables. Since the distributions of the indices were not normal, associations between different quality indicators, between quality indicators and dependent variables (illness-related variables), and between quality indicators and patients’ background characteristics were calculated using bivariate Spearman’s rho. In case of missing values on indicators, list wise deletion was used in calculations. Linear regression was conducted using quality indicators as continuous predictor variables and the proportion of ill days in the observation period as outcome variable.

## Results

In Table 1, the sample of patients is characterised with demographic and clinical information.

**Table 1:** Sample characteristics at baseline of 137 bipolar patients

Demographic variables	Clinical variables		
	Mean ± SD	Type BD disorder	Total (%)
Age, years	45.9 ± 10.2	Bipolar I	90 (65.7)
		Bipolar II	47 (34.3)
	Total (%)		Mean ± SD
Gender		Duration bipolar disorder, years	21.7 ± 11.5
Male	72 (53)		
Female	65 (47)		
		Onset age bipolar disorder, years	24.1 ± 9.9
Marital status			
With partner	67 (49)	No. of episodes	
Without partner	69 (50)	Depression	15.3 ± 23.4
Unknown	1 (1)	(Hypo)mania	13.8 ± 21.5
Annual income		N of hospitalisations	
<20,000 €	82 (62)	Depression	0.9 ± 2.1
≥20,000 €	50 (38)	(Hypo)mania	1.3 ± 2.3
Educational level			
≤High school	65 (47)		
>High school	71 (52)		
Unknown	1 (1)		

The average age in the sample was 45.9 years (SD 10.2), 53 % was male, 49 % had a partner, 62 % had an income lower than 20,000 euros and 47 % had an education below high-school level. Table 2 provides descriptives of the quality indicators.

**Table 2:** Descriptives of quality indicators

Indicator	Compliance		Consistency			Reactivity
Operationalisation	Average observation length (SD)	Average % missings (SD)	Average Spearman’s rank order correlation LC depression-CGI expert (SD)	Average Spearman’s rank order correlation LC mania-CGI expert (SD)	Average Spearman’s rank order correlation LC mood-LC severity (SD)	Average alcohol consumption week 1 minus week 4 (SD)
Raw average	347 days (90) range: 60–529	1.6 (4.2)*	rho = 0.61 (0.35) N = 122***	rho = 0.63 (0.45) N = 105***	rho = 0.70 (0.28) N = 134	Mean difference no. of drinks 0.15 (1.59)** range: −3.71 to 11.29
Quality indicator average (between 0 and 1)	0.61 (0.19)	0.98 (0.04)	0.80 (0.17)	0.82 (0.22)	0.85 (0.14)	0.74 (0.11)

Number of cases is 137, unless stated otherwise

* N = 48 (35 %) had missing data points

** 51 patients had more drinks in week 4 than in week 1, with a maximum of 3.71 more (sum: 44.29). Also, 51 patients drank less in week 4, but with a maximum of 11.29 less (sum: 64.98). Thus, the difference in a reactive direction—drinking less as a result of reporting alcohol consumption—is higher

*** No correlations could be established in cases where one of the two series of values was constant; this was often the case

In the top row, raw scores are presented; in the second row descriptives of the transformed indicators, ranging from 0 to 1. The least variation was found on the indicators ‘missings percentage’ and ‘reactivity’. Only 35 % of the sample had missing data points during the entire observation period, and the average percentage was low. With regard to reactivity in alcohol consumption, the differences in average number of drinks between weeks 1 and 4 were normally distributed—about equal numbers of patients decreased or increased their alcohol consumption in the first 4 weeks. Of the forty respondents who scored below the average of 0.74 on the reactivity indicator, twelve increased their drinking in weeks 5–7. Hence, it can be argued that clear-cut reactivity is not present in this sample with regard to self-reported daily alcohol consumption.

**Table 3:** Spearman’s rank order correlations between quality indicators

	Compliance		Consistency			Reactivity
	Observation length	Missings %	LC depression-CGI expert	LC mania-CGI expert	LC mood-LC severity	Alcohol consumption week 1–4
Observation length	1	–	–	–	–	–
Missings %	0.14	1	–	–	–	–
LC depression-CGIBP	0.09	0.05	1	–	–	–
LC mania-CGI-BP	−0.03	0.05	0.53**	1	–	–
LC mood-LC severity	0.01	0.13	0.41**	0.33**	1	
Alcohol consumption week 1–4	0.02	0.07	−0.07	−0.10	−0.04	1

** Significant at level p < 0.01

As can be seen in Table 3, the number of significant correlations between quality indicators was moderate: only three correlations attained significance. They concern correspondences between related constructs: correspondence between patient and clinician judgment about functioning in term of depression and mania, and correspondence between self-rated daily mood and severity scores. Most of the significant relations are found for consistency, demonstrating that consistency is the best indicator. Again, reactivity and missings percentage scored lowest of the six indicators. Associations between quality indicators and four outcome variables—the proportions of ill days in the observation period and the numbers of depressive, manic and hypomanic episodes—are presented in Table 4.

**Table 4:** Spearman’s rank order correlations of quality indicators with dependent variables (illness indicators)

	Observation length	Missings %	LC depression-CGI expert	LC mania-CGI expert	LC mood-LC severity	Reactivity, alcohol
Proportion of ill days	−0.10	−0.10	−0.36**	−0.19*	−0.13	−0.04
No. of depressive episodes	0.02	−0.00	−0.01	0.10	−0.05	−0.00
No. of manic episodes	0.05	−0.03	0.09	−0.10	0.02	−0.05
No. of hypomanic episodes	0.09	−0.14	−0.28**	−0.50**	−0.11	0.04

* Significant at level p < 0.05

** Significant at level p < 0.01

Seventeen of the twenty-four correlations have negative signs, indicating that a lower data quality obtains when values for the dependent variables (indicators of illness) increase. Only four significant correlations are found on the outcome variables, ranging from −0.19 to −0.50 (4–25 % variance explained). The higher the scores on quality indicators, the smaller the number of ill days and ill episodes experienced; lower data quality obtains for patients with relatively large numbers of ill days and manic or depressive episodes. A regression analysis was performed on the number of ill days, using the quality indicators as predictors after natural log transformation; the results are shown in Table 5.

**Table 5:** Regression of quality indicators on number of ill days

	Standardised beta	*p* value
Observation length	−0.23	0.02
Missings %	−0.10	0.33
LC depression-CGI-BP	−0.29	0.02
LC mania-CGI-BP	−0.09	0.37
LC mood-LC severity	0.11	0.31
Reactivity, alcohol	−0.07	0.49
Adjusted R square: 0.11		

Five of the six betas are negative; the higher the value of the data quality indicator, the lower the number of ill days that the patient experiences. Only two betas (missings % and LC depression-CGI-BP) reach significance.

Finally, relations between quality indicators and background characteristics are presented in Table 6.

**Table 6:** Spearman’s rank order correlations of quality indicators with background characteristics

	Compliance		Consistency			Reactivity
	Observation length	Missings %	LC depression-CGI expert	LC mania-CGI expert	LC mood-LC severity	Alcohol consumption week 1–4
Education	0.04	0.06	−0.07	−0.11	0.00	0.03
Income	0.14	0.10	0.03	−0.03	0.01	−0.19*
Age	0.09	0.03	0.21*	0.13	0.11	−0.24*
Gender (t-test)	t = 2.55 p = 0.012^#^	t = −0.59 NS.	t = 0.84 NS	t = −0.63 NS	t = 0.97 NS	t = 1.52 p = NS

* Significant at level p < 0.05

^#^Positive T: for females a higher data quality obtains

Positive correlations in Table 6 indicate higher data quality. For example, older persons have higher values on the ‘correlation between LC depression and clinician judgment depression’ quality indicator. People with lower incomes and younger people are relatively sensitive to reactivity with regard to alcohol consumption.

## Conclusion

We have found weak effects: up to twenty-five per cent explained variance of the constructed quality indicators for daily prospective self-ratings concerning disease course. The main conclusion is that the more affected the patient is in terms of experienced number of ill days and manic and depressive episodes, the lower the data quality will be.

## Discussion

The results could be interpreted as follows: it is sensible to handle self-ratings of rather ill respondents with caution, as patients heavily affected by mood symptoms are ‘worse daily raters’. Researchers should be aware of the fact that the more severe patients’ bipolar disorder is, the less valid or reliable self-ratings will be. The effects found in terms of explained variance are low to moderate: this may on the one hand indicate that the quality of this dataset is adequate; on the other hand, the indicators are informative, since they do explain some variation.

This specific sample may have had some characteristics that influenced the results: patients were rather compliant in filling out the LCM, which is expressed in low percentages for missing data points. The average disease length was about 22 years (Table 1), which is rather long. Patients in this sample have a low chance of recovery; their situation can best be described as ‘stable’. Since most members of the sample were also adherent to therapy and motivated (48 patients were members of the DAMDR), on entering the study a self-selection effect may have occurred. This means that in other samples the compliance indicators may score worse and may have stronger relations to disease outcome variables. New developments in life charts for bipolar patients involve electronic recording via the web. This electronic data collection instrument provides the option of monitoring the exact dates and times of recording. The result that the association between self rating and clinician CGI rating of depression are negatively related to illness severity has a clinical implication: the more patient and clinician agree with respect to depression ratings, the smaller the number of self reported ill days. Also, when it is difficult to obtain self ratings for a long observation period, this can be an indication that the patient is severely ill.

Limitations of this study concern the fact that data quality indicators were newly developed: no existing operationalisations were available. For example, it is not clear whether the indicator reactivity, a reported change in alcohol use consumption in the first four weeks represents a real change in sensitive behaviour or underreporting of the actually consumed number of alcohol units. However, in a previous publication [3] we also observed that a subgroup of heavy drinking patients in the sample studied, significantly reduced their alcohol consumption in the follow up year as compared to the first four weeks of the observation period. This may indicate a real behaviour change in the long term. In this study we have chosen to use only the first four weeks of the observation period to assess immediate reactivity to self reporting of alcohol consumption. Replications of the use of comparable operationalisations can give more insight in the use of such indicators. Lastly, to establish inter-rater reliability of the ratings of patient functioning ratings of different clinicians would have been necessary. Yet this was impossible due to restrictions on costs and logistics of the study.
